# Effects of vitamins C and E in tris citric acid glucose extender on chilled semen quality of Kail ram during different storage times

**DOI:** 10.1038/s41598-023-43831-2

**Published:** 2023-10-23

**Authors:** Shereen Akhter, Muhammad Zubair, Majid Mahmood, Syed Murtaza Hassan Andrabi, Nasir Hameed, Ejaz Ahmad, Muhammad Kashif Saleemi

**Affiliations:** 1 Department of Zoology, University of Poonch Rawalakot, Rawalakot, Pakistan; 2 Department of Veterinary Clinical Sciences, University of Poonch Rawalakot, Rawalakot, Pakistan; 3 The Animal Sciences Institute, NARC, Islamabad, Pakistan; 4 Department of Clinical Sciences, Faculty of Veterinary Sciences, Bahauddin Zakariya University, Multan, Pakistan; 5 Department of Pathology, University of Agriculture Faisalabad, Faisalabad, Pakistan

**Keywords:** Biotechnology, Cell biology

## Abstract

Mammalian sperm cells are highly vulnerable to lipid peroxidation by free radicals. Antioxidants such as vitamin E, and vitamin C neutralize the activity of free radicals and protect the sperm from reactive oxygen species. The present study was conducted to investigate the effects of vitamin C, vitamin E, and their combination in a Tris-based extender on the semen quality of Kail Ram. Semen samples from five mature Kail rams were collected in this study. The semen samples were diluted by Tris-glucose-egg yolk. Diluted semen samples were divided into four parts. The first part was added with 1 mg/ml of vitamin C, the second part was added with 1 mg/ml of vitamin E and the third part was added with both vitamin C and E, in combination with a dose of 1 mg/ml. The fourth part was considered as control without any addition. The diluted semen samples were cooled gradually and preserved at 5˚C for three days. Sperms in chilled diluted semen samples were examined for motility, viability, and plasma membrane integrity every 24 h for three days (72 h). Present study results showed significant (P < 0.05) effects of vitamins C, E, and their combination on some parameters such as motility, straightness of average special path, linearity of the curvilinear trajectory, and beat-cross frequency. However, there was no significant (P < 0.05) effect of storage duration and antioxidants (vitamin C, E, and their combination) on viability, sperm plasma membrane integrity, and some CASA parameters. From the present study, it could be concluded that the supplementation of vitamins C, and E, and their combination do not enhance the life span and quality of semen in Kail ram during liquid storage at 5 °C.

## Introduction

Artificial insemination (AI) is considered a major tool of genetic propagation to enhance reproduction efficiency in different species of animals^1^. Semen preservation plays a vital role in artificial insemination. For semen storage, two basic techniques are: (1) chilling, and (2) Cryopreservation^2^. In the sperm plasma membrane of ram huge amounts of polyunsaturated fatty acids are present. A strong antioxidant system is absent in ram sperm and seminal plasma. Due to the lack of an efficient antioxidant system, ram sperms are highly vulnerable to cryopreservation, which reduces semen cryopreservation in sheep^3^. Alternatively, insemination with diluted fresh and chilled semen has been adopted to improve the pregnancy rate. However, insemination with chilled semen can only be done over a short period of time^4^. To increase the storage duration and semen quality, several techniques have been developed with the addition of extenders (egg yolk, Tris-citrate-fructose, Tris- and Tris-glucose-egg yolk). Semen extenders are used to protect sperm from harmful effects like freeze and osmotic shock, cooling stress, cell injury by ice crystals, and oxidative stress. Semen extenders enhance or maintain sperm integrity and provide a favorable pH and ATP^2^. Extender is a chemical medium that is used to extend the storage period of semen. Semen extenders protect sperm against different shocks during the processing and transportation of semen samples. Also, it is a source of energy for metabolic activities within sperm cells. To protect semen from microbial growth extenders also monitor the contamination of medium^5^.

Different types of antioxidants such as enzymes, extracts from some plants, vitamins (vitamins C, E, and B12), Olive oil, nanoparticles, and Glutathione effectively improve semen parameters^6^. Antioxidants are used to prolong the storage period of semen, improve the acrosomal integrity, and fertilization capacity of sperm, reduce the degree of sperm cell damage, enhance or maintain viability, and motility^7^.

The most important method to enhance or maintain the motility and viability of ram sperm cells is the supplementation of vitamins C and E. Treatment of sperm with antioxidants reduces the frequency of DNA damage^8^. Antioxidants such as vitamins E, and C neutralize the activity of free radicals and protect the sperm from reactive oxygen species^9^. Vitamin E is a lipid-soluble, and the major chain-breaking antioxidant that supports the mechanism of cell defense. Vitamin E donates the Hydrogen from the Hydroxyl group to free radicals and makes them unreactive. The supplementation of vitamin E as an antioxidant to semen extender inhibits lipid peroxidation caused by ROS and thus prevents sperm motility^10^.

Vitamin C is a nonenzymatic, water-soluble antioxidant. It is an effective reactive oxygen species with high vitality. Vitamin C as an antioxidant can affect the performance of sperm by reducing sperm cell damage through its radical forager activity. It is important to improve sperm motility and viability or to provide a protective effect against DNA sperm damage^11^.

Although much work has been done through the supplementation of vitamins C and E to cattle semen. However, limited information is available for their use in Kail ram semen. So, we hypothesized that supplementation of antioxidants to tris-based extender will improve the semen quality in Kail ram. The current study was designed to evaluate the effects of vitamin C, vitamin E, and their combination on the quality of chilled Kail Ram semen in tris-based extender during different periods.

## Materials and methods

The study was conducted at Livestock Research Farm Khaigala, University of Poonch Rawalakot. Adult ram (n = 5) with a body condition score (BCS) ranging from 2.5 to 4.5 on a scale of 1 to 5 (1 = emaciated, 5 = obese) were selected for semen collection. The rams were grazing on natural grazing land for 5 to 6 h daily. They were provided free excess to safe drinking water thrice a day and dewormed routinely. They were housed at night and grazed during the day with the addition of commercial concentrate ICI vanda, and maize grain 200 g/day each.

### Semen collection and dilution

The study was approved by the "Human and animals ethics committee", University of Poonch Rawalakot, all the experiments were performed according to relevant guidelines and complied with the ARRIVE guidelines. All the rams were separated from the sheep into a separate animal shed. All five rams were for semen collection into an artificial vagina. The temperature of water in the inner liner of the artificial vagina was kept from 42 to 45 °C. Semen was collected twice a week i.e. Monday and Thursday. About 30 ejaculations were collected from 5 rams. A total of six pooled ejaculates (n = 6) were used in the current experiment. The pooled semen was divided into four equal parts and diluted with an equal volume of extender (1:1) and then more extender was added within 15 min to make the final dilution 1:7. Furthermore, each aliquot with a specific concentration of Vitamin C, E, and their combination was stored at 5 °C for 3 days. The temperature of aliquots was reduced slowly to 5 °C at the rate of −0.3 °C/ min. After collection, the semen was stored immediately at 37 °C temperature in a water bath to evaluate the quality parameters of the fresh semen. A tris-based extender was used in the current experiment. The Tris-based extender (Tris) was prepared with 80 ml of Tris citric acid fructose buffer (Tris hydroxymethyl amino methane—3. 028 g, citric acid monohydrate—1.70 g; glucose—1.25 g; distilled water ad—100 ml) with 20 ml of egg yolk. The Tris-based extender was supplemented with vitamin C 1 mg/ml, Vitamin E 1 mg/ml, and a combination of both vitamin C and vitamin E.

### Addition of vitamins C, E and their combination

After dilution semen sample was divided into four parts. The first part of the semen sample was supplemented with a dosage of 1 mg/ml vitamin C, the second part was supplemented with a dosage of 1 mg/ml vitamin E, and the third part was supplemented with a combination of vitamin C and E. Fourth part of semen samples were considered as a control without addition of any antioxidant. After dilution semen samples were cooled gradually and stored at 5 °C for 3 days. Cooled diluted semen samples were examined for individual sperm motility, viability, and plasma membrane functional integrity every 24 h for 3 days.

### Semen evaluation

Sperm motility and Kinematics were evaluated using the motility and concentration module of SCA^®^ software, version 6.2.0.1 (Microptics S. L., Barcelona, Spain) using the following setup: temperature 37 °C; particle area: 20 to 60 μm^12^; drifting: 10 μm/s; slow medium: 5 μm/s; rapid > 25 μm/s; progressivity > 80% of STR; VAP points: 5; connectivity: 10. Briefly, 5 µL of fresh diluted semen was placed on a pre-warmed (37 °C) glass slide and evenly spread by placing cover slip to evaluate motility and kinematics Curvilinear velocity (VCL), Average path velocity (VAP), Straight line velocity (VSL), Amplitude of lateral head displacement (ALH), Straightness of the average special path (STR), Linearity of the curvilinear trajectory (LIN), Oscillation index value (WOB) and Beat-cross frequency (BCF) parameters in five fields.

### Sperm plasma membrane functional integrity

To measure the membrane functional integrity of ram sperm, 500µL solution of hypo-osmotic swelling (HOS) solution (0.735 g of tri-sodium citrate dihydrate and 1.351 g fructose) of 75 mosm/kg was incubated with 50 µL of semen in a water bath at a temperature of 37 °C for 30 min. A small drop of incubated semen was observed under a phase-contrast microscope (BX51, Olympus, Japan) at 400× magnification and two hundred sperm were counted for swelling/cooling of the tail. The sperm with coiled tails were considered functionally intact.

### Sperm viability

The sperm viability (live-dead) was studied by using an eosin-nigrosin stain under a phase-contrast microscope (BX51, Olympus, Japan) at 400 × magnification. The stain was prepared by mixing 3 g of sodium citrate dehydrate with 100 ml of double distilled water. Then 1 g of Eosin and 5 g Nigrosin were added and mixed well with the help of a stirrer. This stain has the principle that Eosin dye stains the dead sperm a pink color, while the live sperm remains colorless. The Nigrosin provides a black-blue background.

### Data analysis

The data were analyzed by using SPSS (16 Version) software package. The normality of data was checked through the Shapiro–Wilk test. The effects of vitamin C and E and storage temperature on semen quality parameters were analyzed using repeated measure ANOVA. Bonferroni posthoc tests were performed to find out the significant differences in semen parameters. Significance was assigned at p < 0.05.

### Ethical approval

The rams kept in this experiment were approved from Ethical approval of University of Poonch Rawalakot.

### Consent to participate

It is hereby declared that all the authors have consent for editing in this manuscript.

## Results

### Effect of antioxidants and storage time on sperm motility parameters (%)

Three treatment groups and storage time had a significant (P < 0.05) effect on sperm motility. The percentage of sperm motility was similar (P > 0.05) for the three antioxidants after 24 h storage (Table 1). After 72 h of storage, treatment group C + E had higher (P < 0.05) sperm motility (66 ± 4.6), progressive motility (21 ± 3.9), rapid (2.6 ± 2.1), and medium progressive motility (18.5 ± 4.8) compared with vitamin C (51 ± 2.1, 12.4 ± 2.8, 0.0 ± 0.6, 2.4 ± 2.6), vitamin E (57 ± 0.7, 17 ± 6.3, 2.3 ± 0.3, 13.8 ± 10.8) and control group (53 ± 3.5, 15 ± 3.9, 0.7 ± 0.1, 14.8 ± 3.8), respectively (Table 1). Sperm storage time had a significant effect on sperm motility parameters. Reversely with the increasing storage time sperm motility, progressive motility, and rapid, and medium progressive motility decreased. Furthermore, storage time negatively affected motility parameters for all three treated groups (Table 1).

**Table 1:** Effect of supplementation of Vit. E and C to tris-based extender on chilled semen quality of Kail rams.

Parameters	Groups	Time (h)				*P*-value		
		0	24	48	72	*G*	*T*	G*T
Motility (%)	A	83 ± 5.4^A^	76 ± 2.7^AB^	72 ± 4.8 ± ^AB^	53 ± 3.5^B^	0.007	0.000	0.000
	B	86 ± 1.7^A^	76 ± 1.9^AB^	65 ± 5.2^AB^	51 ± 2.1^C^			
	C	78 ± 2.5^A^	74 ± 7.0^AB^	61 ± 0.9^AB^	57 ± 0.7^C^			
	D	82 ± 4.2^A^	70 ± 6.9^A^	78 ± 5.9^A^	66 ± 4.6^A^			
PM (%)	A	42 ± 6.4^A^	30 ± 3.4^AB^	26 ± 2.3^AB^	15 ± 3.9^C^	0.694	0.000	0.183
	B	47 ± 3.9^A^	26 ± 3.4^BC^	25 ± 3.0^CB^	12.4 ± 2.8^D^			
	C	40 ± 2.3^A^	31 ± 3.8^AB^	25 ± 7.7^AB^	17 ± 6.3^C^			
	D	34 ± 3.8^A^	25 ± 2.1^AB^	26 ± 5.9^AB^	21 ± 3.9^B^			
RP (%)	A	5.2 ± 0.7^A^	3.1 ± 1.0^AB^	2.4 ± 0.7^AB^	0.7 ± 0.1^C^	0.378	0.003	0.132
	B	4.6 ± 1.0^A^	2.4 ± 0.9^AB^	1.9 ± 0.8^AB^	0.0 ± 0.6^B^			
	C	3.9 ± 0.4^A^	3.4 ± 0.8^A^	3.3 ± 0.4^A^	2.3 ± 0.3^A^			
	D	3.1 ± 0.6^A^	2.8 ± 0.6^A^	4.4 ± 2.3^A^	2.6 ± 2.1^A^			
MP (%)	A	37.4 ± 5.9^A^	27.7 ± 2.4^AB^	24.1 ± 2.0^AB^	14.8 ± 3.8^B^	0.466	0.000	0.361
	B	42 ± 3.1^A^	22.0 ± 2.8^BC^	24 ± 3.3^CB^	2.4 ± 2.6^D^			
	C	36.1 ± 2.3^A^	35.7 ± 3.8^AB^	27.7 ± 12.2^AB^	13.8 ± 10.8^B^			
	D	31.3 ± 3.9^A^	24.9 ± 7.5^A^	19.6 ± 5.8^A^	18.5 ± 4.8^A^			

Data were expressed as mean ± SEM.

Group A = control; Group B = vit. C 1 mg/ml; Group C = vit. E 1 mg/ml; Group D = vit. C + E; *G* group, *T* storage time, G*T group storage time interaction, *PM* progressive motile sperm, *RP* rapid progressive sperm, *MP* medium progressive sperm.

Different superscripts ^ABC^ within a row indicates significant difference (P < 0.05) within a specific group at different time intervals.

### Effect of antioxidants and storage time on sperm kinematic parameters (%)

The sperm curvilinear velocity (VCL) was similar(P > 0.05) for control, vitamin C and C + E groups after 24 and 48 h of storage at 5 °C. While the vitamin E group had a similar (P > 0.05) VCL percentage up to 72 h (Table 2). The average path velocity (VAP) was similar(P > 0.05) for the vitamin C and E treated group after 24 and 48 h. While for control and Vitamin C and E combination VAP was similar (P > 0.05) up to 72 h. The control group had a higher VAP percentage after 48 h compared with Vit. C, E, and their combination (Table 2).

**Table 2:** Effect of supplementation of vit. E and C to tris-based extender on chilled semen quality of Kail rams.

Parameters	Groups	Time (h)				*P*-value		
		0	24	48	72	*G*	*T*	G*T
VCL	A	54.4 ± 4.8^A^	48.2 ± 3.8^AB^	42.2 ± 2.2^AB^	40.1 ± 2.2^C^	0.054	0.000	0.765
	B	50.1 ± 2.6^A^	39.1 ± 3.9^AB^	37.9 ± 8.2^AB^	30.8 ± 7.6^C^			
	C	51.6 ± 2.9^A^	50.4 ± 1.9^A^	42.7 ± 2.2^A^	41.8 ± 3.2^A^			
	D	46.6 ± 5.9^A^	43.2 ± 2.7^AB^	42.6 ± 3.2^AB^	33.2 ± 3.9^A^			
VAP	A	29.4 ± 2.7^A^	27.8 ± 1.2^A^	24.5 ± 0.7^A^	24.1 ± 1.6^A^	0.219	0.000	0.306
	B	27.9 ± 2.0^A^	23.3 ± 1.8^AB^	23.1 ± 2.2^AB^	18.2 ± 1.7^B^			
	C	29.5 ± 1.3^A^	27.2 ± 0.3^AB^	23.6 ± 3.9^BC^	18.4 ± 5.3^A^			
	D	26.1 ± 2.3^A^	25.4 ± 1.3^A^	21.3 ± 1.9^A^	17.6 ± 1.4^A^			
VSL	A	17.4 ± 1.9^A^	15.9 ± 0.9^AB^	14.5 ± 0.4^AB^	13.3 ± 0.5^C^	0.169	0.005	0.708
	B	15.9 ± 0.6^A^	15.2 ± 0.5^AB^	14.9 ± 0.9^AB^	12.8 ± 1.2^B^			
	C	17.3 ± 1.0^A^	15.8 ± 1.1^A^	15.7 ± 0.9^A^	15.8 ± 0.7^A^			
	D	15.4 ± 0.9^A^	15.6 ± 0.6^A^	14.8 ± 1.1^A^	14.4 ± 0.8^A^			
STR	A	60.8 ± 1.6^A^	58.7 ± 2.1^A^	56.8 ± 2.2^A^	22.0 ± 1.7^A^	0.025	0.187	0.136
	B	58.7 ± 4.3^A^	70.9 ± 7.2^AB^	69.1 ± 9.1^AB^	67.7 ± 3.2^B^			
	C	60.7 ± 0.9^A^	68.3 ± 5.4^A^	58.3 ± 2.8^A^	56.9 ± 7.0^A^			
	D	62.4 ± 2.7^A^	72.6 ± 4.1^AB^	64.0 ± 3.4^AB^	62.0 ± 3.8^A^			

Data were expressed as mean ± SEM.

Group A = control; Group B = vit. C 1 mg/ml; Group C = vit. E 1 mg/ ml; Group D = vit. C + E; *G* group, *T* storage time, G*T group storage time interaction, *VCL* curvilinear velocity, *VAP* average path velocity, *VSL* straight line velocity, *STR* straightness of average special path.

Different superscripts ^ABC^ within row indicates significant difference (P < 0.05) within a specific group at different time intervals.

Vitamin C and the control group had similar (P > 0.05) straight line velocity (VSL) after 24 and 48 h of storage. While both vitamin E and C + E had similar (P > 0.05) VSL percentages up to 72 h (Table 2). For the control and vitamin E-treated group, the straightness of the average special path (STR) was similar (P > 0.05) up to 72 h of storage. Vitamin C and a combination of Vit C and E had similar STR % after 24 h. However, vitamin C had a higher STR percentage up to 72 h (67.7 ± 3.2) compared with vitamin E, C + E, and control groups. Furthermore, storage time had a negative impact on sperm kinematic parameters in all three antioxidants and the control group (Table 2).

### Effect of antioxidants and storage time on LIN, WOB, ALH, and BCF (%)

The control group and vitamin E had similar (P > 0.05) linearity of the curvilinear trajectory (LIN) percentages up to 72 h of storage at 5 °C. However, the vitamin C and C + E treated group had similar LIN percentages after 24 and 48 h. Furthermore, after 72 h vitamin C had higher LIN (45.4 ± 3.4) compared with the control, vitamin E, and C + E treated group (Table 3). The effect of storage time and all three antioxidants, vitamin C, and E, and their combination was not significant (P > 0.05) on the oscillation index value (Table 3).

**Table 3:** Effect of supplementation of Vit. E and C to tris-based extender on chilled semen quality of Kail rams.

Parameters	Groups	Time (h)				*P* value		
		0	24	48	72	*G*	*T*	G*T
LIN	A	36.6 ± 1.9^A^	40.4 ± 3.6^A^	40.5 ± 1.9^A^	38.4 ± 2.5^A^	0.031	0.023	0.200
	B	37.2 ± 6.6^A^	61.2 ± 5.2^AB^	50.8 ± 14.6^AB^	45.4 ± 3.4^B^			
	C	38.8 ± 1.0^A^	44.1 ± 6.1^A^	41.3 ± 3.7^A^	34.3 ± 1.1^A^			
	D	38.9 ± 4.2^A^	54.8 ± 8.2^AB^	42.8 ± 3.4^AB^	36.6 ± 5.1^A^			
WOB	A	58.0 ± 1.6^A^	64.0 ± 0.1^A^	61.8 ± 3.8^A^	61.0 ± 2.8^A^	0.493	0.114	0.743
	B	59.5 ± 5.4^A^	68.4 ± 11.3^A^	65.7 ± 9.8^A^	64.0 ± 2.1^A^			
	C	60.8 ± 1.3^A^	63.5 ± 5.4^A^	58.1 ± 3.1^A^	57.9 ± 4.7^A^			
	D	59.9 ± 3.4^A^	71.5 ± 7.3^A^	63.6 ± 2.3^A^	56.5 ± 5.2^A^			
ALH	A	2.9 ± 0.1^A^	2.7 ± 0.1^A^	2.4 ± 0.1^A^	2.4 ± 0.1^A^	0.062	0.001	0.821
	B	2.8 ± 03^A^	2.3 ± 0.1^AB^	2.1 ± 0.1^AB^	2.0 ± 0.2^C^			
	C	2.9 ± 01^A^	2.9 ± 0.2^A^	2.5 ± 0.1^A^	2.4 ± 0.3^A^			
	D	2.6 ± 02^A^	2.6 ± 0.2^AB^	1.9 ± 0.2^AB^	2.5 ± 0.4^A^			
BCF	A	4.8 ± 0.4^A^	4.2 ± 0.5^AB^	3.9 ± 0.2^AB^	2.8 ± 0.4^B^	0.014	0.001	0.087
	B	4.5 ± 0.2^A^	3.9 ± 0.4^AB^	3.9 ± 0.1^AB^	1.4 ± 1.5^B^			
	C	4.7 ± 0.5^A^	4.4 ± 0.5^A^	4.4 ± 0.3^A^	4.3 ± 0.3^A^			
	D	4.2 ± 0.4^A^	4.9 ± 0.9^A^	4.1 ± 0.4^A^	4.0 ± 0.7^A^			

Data were expressed as mean ± SEM.

Group A = control; Group B = vit. C 1 mg/ml; Group C = vit. E 1 mg/ml; Group D = vit. C + E, *G* group, *T* storage time, G*T group storage time interaction, *LIN* linearity of the curvilinear trajectory, *WOB* oscillation index value, *ALH* amplitude of lateral head displacement, *BCF* beat-cross frequency.

Different superscripts ^ABC^ within a row indicates significant difference (P < 0.05) within a specific group at different time intervals.

The control group and vitamin E had similar percentages of the amplitude of lateral head displacement (ALH) up to 72 of storage at 5 °C. While, vitamin C and Vit. C + E treated group had similar ALH percentages after 24 and 48 h. However, after 72 h vit. C + E treated group had higher ALH (2.5 ± 0.4) than the control, vitamins C and E (Table 3). Furthermore, the percentage of beat cross frequency (BCF) was similar (P > 0.05) up to 72 for vit. C and Vit. C + E treated group. For vitamin C and the control group, BCF was similar after 24 and 48 h. However, after 72 h control group had higher (2.8 ± 0.4) BCF than Vit. C (1.4 ± 1.5) but the difference was not significant between them (Table 3).

### Effect of antioxidants and storage time on plasma membrane integrity and viability (%)

The effect of the three antioxidants vitamin C, E, and the combination of vitamin C and E was not significant (P > 0.05) on sperm plasma membrane integrity and viability (Table 4). In addition, the impact of sperm storage time was also not significant (P > 0.05) on plasma membrane integrity and viability (Table 5).

**Table 4:** Comparison of percentage HOST and viability of different treatments.

Parameters	Group A	Group B	Group C	Group D	*P* value
HOST	67.7 ± 11.4	73.1 ± 8.5	72.3 ± 10.6	70.5 ± 7.8	0.527
Viability	64.6 ± 17.8	61.2 ± 13.7	57.1 ± 13.8	53.4 ± 10.6	0.927

Group A = control; Group B = vit. C 1 mg/ ml; Group C = vit. E 1 mg/ml; Group D = vit. C + E.

**Table 5:** Comparison of percentage HOST and viability on different time durations.

Parameters	0 h	24 h	48 h	72 h	*P *value
HOST	71.9 ± 5.6	74.5 ± 8.0	65.6 ± 12.0	61.6 ± 11.5	0.059
Viability	67.2 ± 15.0	62.3 ± 16.6	58.4 ± 10.1	54.6 ± 10.5	0.255

Group A = control; Group B = vit. C 1 mg/ml; Group C = Vit. E 1 mg/ml; Group D = Vit. C + E.

## Discussion

In mammals, the sperm plasma membrane has a large amount of polyunsaturated fatty acids which lead to reduce the sperm motility^7^. Vitamin C and E are known antioxidants to enhance the quality of sperm. These antioxidants are the components of natural defense mechanisms in mammalian semen that provides protection to male reproductive system and improves viability and sperm motility parameters^13,14^.

In the present study, the addition of vitamin C, and vitamin E, and their combination improved sperm motility at 24, 48, and at 72 h of storage at 5 °C, compared with the control group. This is the first of its kind to explore the effects of these vitamins in this particular context, specifically in the evaluation of semen using Microptic CASA in ram. Previously, Donoghue et al.^15^ reported that vitamin E addition to sperm diluent helps to maintain motility up to 75% for 48 h. Similar results were reported by Azawi et al*.* (2013). The study reported that Awassi ram semen supplemented with antioxidants vitamins C, and E can improve sperm motility for 120 h of storage at 5 °C^8^. The addition of vitamin E at the concentration rates of 0.75 mg and 1 mg per ml significantly improved sperm motility^16^. This improvement in sperm motility by vitamin C and vitamin E is due to the antioxidant activity of vitamin C, and E that inhibits the production of free radicals during lipid peroxidation^17^.

In our findings, there was no significant effect of treatment on progressive motility. The findings of the current study are dissimilar to some studies in Merino rams^17^ and bovine^18^. The addition of vitamin E as an antioxidant at a concentration of 3 mM helped in the liquid preservation of semen up to 72 h at 4 °C with higher progressive sperm motility^10^. In the present study, there was no significant effect of antioxidants on rapid progressive motility. In contrary to the current study, ^10^reported that vitamin E with a dosage of 0.06 mg/ml showed higher progressive sperm motility. In addition, the results of another study showed that the addition of vitamin C to the CEY extender increased sperm progressive motility^19^. There was no significant effect of treatment on medium progressive motility. Similar results were reported by some previous studies, that the addition of vitamin E at concentrations of 0.05 mg/ml, 0.016 mg/ml and 0.048 mg/ml do not significantly improve the preservation of sperm motility during storage at 5 °C^7^.

There was no significant effect of antioxidants on the kinematic parameters (VCL, VAP, and VSL) of sperms. In contrast, Minaei et al. (2012) reported that vitamin E analog (trolox) affects the curvilinear velocity of sperms significantly. The combination of vitamin C, and E, create higher sperm kinematic parameters such as sperm curvilinear velocity^20^. Vitamin E analog (trolox) affects the average path velocity of sperms significantly^21^. The findings of the present study are contrary to the findings of ^22^ in ram semen. Castellini et al.^23^ reported similar results using different doses of Vit. E. The addition of Vit. C, in an extender at a dosage of 4.5 mg/ml significantly enhances sperm motility and straight-line velocity^18^. Vitamin C, and E in combination make a significant effect on sperm kinematic parameters such as straight-line velocity. similar results were reported by^20^ in the bull. The antioxidants vitamin C and E protect against lipid peroxidation and improve sperm kinematics^13,14^.

In current findings, there was a significant effect of treatment on the straightness of the average special path, linearity of the curvilinear trajectory, and beat cross frequency. Similar results were reported by some previous studies that the supplementation of vitamin E in chilled semen significantly improves the sperm linearity and straightness of average special path^24^. The combined actions of vitamin C, and E, may have played a fundamental role in improving the linearity of the curvilinear trajectory by reducing the oxidation process^25^. Another study revealed that the higher concentration of vitamin C resulted in a significant decrease in sperm motility parameters^26^. In the current study, there was no significant effect of treatment on the amplitude of lateral head displacement. Contrary results were reported by^18,24^.

The decline in sperm motility and kinematic parameters were observed over storage time, especially after 24, 48, and 72 h. Furthermore, storage time negatively affected sperm parameters for all three treated groups. Previously, Qureshi et al*.* (2013) reported a reduction in sperm motility in liquid storage during the first 24 h after collection^27^. A significant decline in rapid progressive motility was observed over storage time, especially after 72 hours^28^, reported decrease in sperm motility between 24 and 48 h of liquid storage. Extenders supply nutrients and a suitable medium where sperms can survive for 60–72 h or even 96 h for artificial insemination^29,30^. Some previous studies reported that extenders conserve sperm kinematic parameters even up to three days after collection^31^.

Storage temperature, extenders, and procedure of extender preparation can affect semen parameters^32^. Some previous studies demonstrated that semen incubation temperature affects the sperm parameters^33,34^. Some previous studies reported that sperm can survive for 60–72 h or even 96 h ^29,30^. Murphy et al*.* (2018) reported that chilled semen can be preserved for as long as 48 h for insemination with lower pregnancy rates^29^.

In the current study, time duration had no beneficial effect on plasma membrane functional integrity and viability of kail ram semen. Previously, Zhang et al. (2020) reported similar results that the viability and sperm membrane integrity decreased with time at 16 °C^3^. Some previous studies also reported that in Awassi ram the plasma membrane functional integrity and viability can be increased for 120 h of preservation at 5 °C and came in argument with the current results^8^. The possible reason behind the decrease in viability and plasma membrane integrity might be the dose level and source of antioxidants used in this study. Contra wise previous study reported that the addition of vitamin E at 3 mM in tris extender helped in liquid maintenance of buck semen up to 72 h at 4 °C with higher viability and plasma membrane functional integrity^10^.

Antioxidants had no profound effect on sperm plasma membrane integrity and viability of Kail ram semen in the current study. Varisli et al*.* (2003) recommended that ram sperm can be preserved successfully for up to 96 h^35^. Some other previous studies demonstrated that the supplementation of vitamin C (200 µg/mL) and vitamin E (5 µg/mL) can improve the viability of semen. Moreover, the addition of vitamins E and C in high concentration negatively affects semen quality parameters^11^. Asmarawati et al*.* (2010) reported that the addition of vitamin E helps to maintain sperm motility, and viability after 72 h of storage at 4 °C^36^. While, vitamin C, addition resulted in decreased motility, and sperm viability. Some previous studies reported that Vit. C and E can improve sperm viability and sperm plasma membrane functional integrity. The most effective antioxidant to inhibit the lipid peroxidation reaction in the cell membrane is vitamin E^12^. These studies came in argument with the present study results. This damaging effect of antioxidant supplementation on sperm quality parameters might be due to the toxic dosage of vitamins E, and C that destroyed different cellular processes within sperm cells^37^.

Another previous study revealed that vitamin E and vitamin C are the most potent antioxidants in citrate-egg yolk (CEY) extender improving semen quality by reducing lipid peroxidation in sperm cells. While the supplementation of vitamin C in Tris egg-yolk (TEY) extender did not improve sperm viability or motility^19^. Plasma membrane sperm integrity and viability were significantly improved by the dosage of vitamin C at 4.5 mg/ml^18^. Lukusa et al*.* (2019) reported that vitamins C, and E in combination better protected the sperm membranes against reactive oxygen species and lipid peroxidation as compared to the individual addition of vitamin C or E^38^. The effects of Vit. C and E in combination enhanced the sperm parameters to a positive extent. Moreover, the best dosage of vitamins C, and E, in combination to maintain sperm viability, and plasma membrane integrity is (200 + 400 mg/l) to the individual addition of vitamins C, and E. The dosage of vitamin C 200 mg/l and vitamin E 200 mg/l are best for the preservation of semen quality^39^. The effect of both antioxidants Vit. C, and E in combination have been first time studied on Kail ram semen. The results revealed that the addition of vitamin C (1 mg/ml), and E (1 mg/ml) in the semen extender provide no better results. The possible reason behind this might be the concentration of vitamin C and E in combination. The mechanisms of the combined effect are not clear. The individual supplementation of vitamins C and E and their combination need further attention to clarify their optimal concentration levels for sperm protection when used in combination. The present study concludes that supplementation of vitamin C, vitamin E, and their combination, at the dose of 1 mg/ml has no valuable effect on viability and plasma membrane functional integrity of Kail ram semen. However, the supplementation of vitamin C, and vitamin E, and their combination has a beneficial effect on some sperm parameters such as Motility, STR, LIN, and BCF. A significant decrease in sperm parameters was mostly observed after 72 h of storage than the control group at 5 °C. This study needs to investigate deeper into the realm of fertility by conducting trials specially focused on the efficiency of these vitamins, we can gain valuable insights into their potential role in enhancing fertility and improving semen preservation techniques.

## Data Availability

The dataset and the samples for this study are available from the corresponding author upon reasonable request drmuhammadzubair@upr.edu.pk.
